# Chemical Composition and Anti-Urolithiatic Activity of Extracts from *Argania spinosa* (L.) Skeels Press-Cake and *Acacia senegal* (L.) Willd

**DOI:** 10.3390/molecules27133973

**Published:** 2022-06-21

**Authors:** Fatima Ezzahra El oumari, Dalila Bousta, Hamada Imtara, Anissa Lahrichi, Radouane Elhabbani, Ghita El mouhri, Omkulthom Al kamaly, Asmaa Saleh, Mohammad Khalid Parvez, Andriy Grafov, Tarik Sqalli Houssaini

**Affiliations:** 1 Laboratory of Epidemiology and Research in Health Sciences, Faculty of Medicine and Pharmacy, Dental Medicine University of Sidi Mohammed Ben Abdellah, Fez 30070, Morocco; radouane.elhabbani@gmail.com (R.E.); tarik.sqalli@usmba.ac.ma (T.S.H.); 2 Laboratory of Biotechnology, Environment, Agri-Food, and Health (LBEAS), Faculty of Sciences, University of Sidi Mohamed Ben Abdellah, Fez 30050, Morocco; boustadalila@gmail.com; 3 Faculty of Arts and Sciences, Arab American University Palestine, P.O. Box 240, Jenin 44862, Palestine; 4 Laboratory of Biochemistry, Faculty of Medicine and Pharmacy, Dental Medicine Sidi Mohammed Ben Abdellah University, BP 1893, Km 22, Road of Sidi Harazem, Fez 30070, Morocco; anissafmpf@hotmail.fr (A.L.); elm.ghita@gmail.com (G.E.m.); 5 Department of Pharmaceutical Sciences, College of Pharmacy, Princess Nourah bint Abdulrahman University, P.O. Box 84428, Riyadh 11671, Saudi Arabia; omalkmali@pnu.edu.sa (O.A.k.); asali@pnu.edu.sa (A.S.); 6 Department of Pharmacognosy, College of Pharmacy King Saud University, P.O. Box 2457, Riyadh 11451, Saudi Arabia; mohkhalid@ksu.edu.sa; 7 Department of Chemistry, University of Helsinki, P.O. Box 55, FI-00014 Helsinki, Finland; andriy.grafov@helsinki.fi; 8 Department of Nephrology, University of Hospital Hassan II, BP 1835, Atlas, Road of Sidi Harazem, Fez 30700, Morocco

**Keywords:** litholytic activity, calcium oxalate crystallization, optical density, gas chromatograph

## Abstract

Ethnobotanical studies have reported the traditional medicinal uses of *Acacia senegal* (L.) Willd. and *Argania spinosa* (L.) Skeels against kidney stone formation and other chronic kidney diseases. The present work is undertaken to study the litholytic activity and the inhibiting activity of calcium oxalate crystallization by bioactive compounds identified in *Argania spinosa* (L.) Skeels press-cake (residue of Argan oil) and in *Acacia senegal* (L.) Willd. The litholytic activity was studied in vitro on cystine and uric acid stones using a porous bag and an Erlenmeyer glass. The study of the inhibiting activity of calcium oxalate crystallization, was based on temporal measurements of the optical density, registered at a 620 nm wavelength for 30 min using an ultraviolet–visible spectrophotometer. The silylation method was performed to identify phytochemicals, followed by gas chromatography coupled with mass spectrophotometry (GC/MS) analysis. The results show significant litholytic activity of *Argania Spinosa* press-cake hydro-ethanolic extract on uric acid and cystine stones, respectively, with dissolution rates (DR) of 86.38% and 60.42% versus 3.23% and 9.48% for the hydro-ethanolic extract of *Acacia senegal* exudate. Furthermore, the percentages of nucleation inhibition are 83.78% and 43.77% (*p* ˂ 0.05) for *Argania spinosa* and *Acacia senegal*, respectively. The results point to the detection of 17 phytochemicals in *Argania spinosa* press-cake extract, the majority of which are phenolic acids and have potent anti-urolithiatic action.

## 1. Introduction

Urolithiasis, a recurrent disorder, is one of the oldest worldwide urological health problems, characterized by the formation of calculi or stones in the urinary system. Several factors are involved in the etiology of this disease such as dietary habits, dehydration, and some infectious bacteria. Urinary stones are classified according to their chemical composition [1], there are calcium oxalate stones, which are the most prevalent and comprise 80% of urinary stone cases; struvite stones, which comprise 10%; uric acid stones, which comprise 9%; and cystine stones, which comprise 1% [2].

Calculi can be removed from the urinary system using several conventional techniques including extracorporeal shock-wave lithotripsy (ESWL), percutaneous nephrolithotomy (PCNL), retrograde intrarenal surgery (RIRS), and laparoscopic ureterolithotomy. These procedures are very costly and do not prevent recurrence. In addition, they cause side-effects such as acute kidney injury and discomfort [2]. Prescribed drugs such as citrate, thiazide diuretics, and urinary alkalinizers present significant side-effects such as dizziness, weakness, and gastrointestinal upset [3].

*Argania spinosa* (L.), an endemic tree of southwestern Morocco, belongs to the Sapotaceae family [4]. Oil extracted from *Argania spinosa* (L.) fruits plays an important socioeconomic role in Amazigh women. Each argan fruit contains two to three kernels, which are used to produce argan oil; unroasted kernels produce cosmetic oil, and roasted kernels produce culinary oil [5]. Regarding the bioactive components of the *Argania spinosa* (L.) tree, *Argania Spinosa* (L.) press-cake remains a diverse source of bioactive phytochemicals (phenolic compounds) [6], and is the residue obtained after argan oil extraction. Several studies have shown that the components of argan press-cake exhibit free-radical scavenging, anti-inflammatory activities and a de-pigmenting effect [7,8,9,10].

Gum arabic is a dried exudate obtained from *Acacia senegal* (L.) trunk and branches. It is used in folk medicine as a natural remedy for kidney stones. Several studies have highlighted its antioxidant effect and its nephroprotective effect against nephrotoxicity and chronic kidney diseases [11,12]. The anti-urolithiatic properties of many phytochemicals such as phenolic acids and flavonoids are well-known. Succinic acid has been found to be effective against renal cancer cell lines. In addition, it exhibits a nephroprotective effect against renal ischemia reperfusion injury [13,14]. Catechin’s antiurolithiatic effect on stone formation may be due to its ability to prevent renal papillary calcification. Catechin was also found to reduce the density of crystals generated by a melamine-cyanuric acid mixture in another investigation [12]. Other phenolic acids such as gallic and tannic acids have been found to be effective against Cisplatin-induced nephrotoxicity [15].

Ethnobotanical studies have reported the traditional medicinal uses of *Acacia senegal* (L.) Willd (also named gum arabic) and *Argania spinosa* (L.) Skeels against kidney stone formation and other chronic kidney diseases [4,5]. Thus far, no studies have confirmed the effectiveness of these two medicinal plants against kidney stone formation. In this context, the purpose of the present study was to determine the chemical compounds found in extracts from *Argania spinosa* (L.) Skeels press-cake and *Acacia Senegal* (L.) Willd, and to evaluate their role in anti-urolithiatic activity using the litholytic-activity method and the anti-crystallization method.

## 2. Results

### 2.1. Analysis of Sample Stones for Litholytic Activity Using Fourier Transform Infrared Spectroscopy Analysis

**Figure 1:**
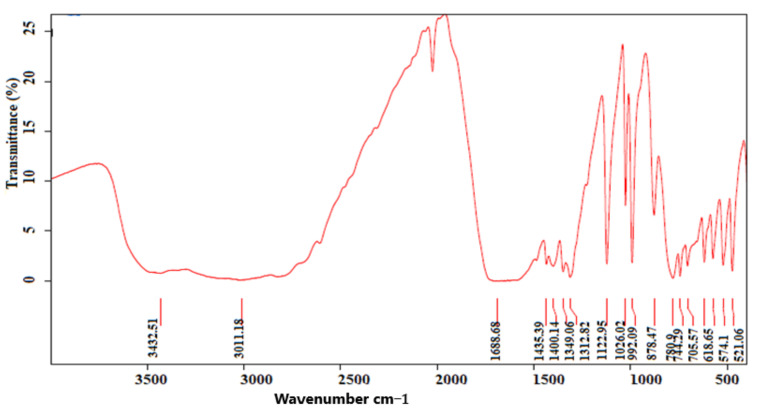
FT−IR spectrum of uric acid stones (FT−IR: Fourier transform infrared spectroscopic technique).

**Figure 2:**
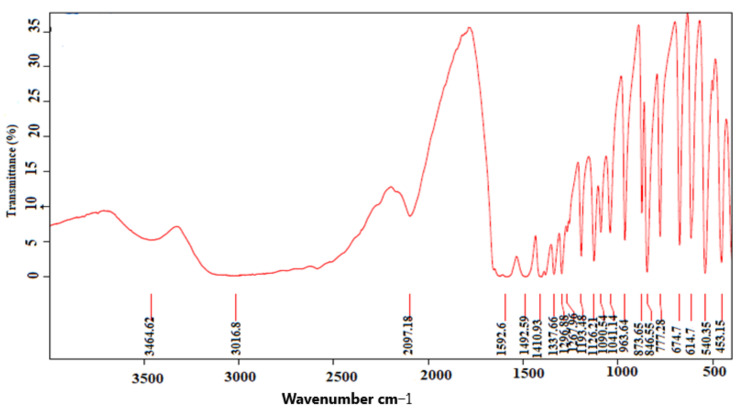
FT−IR spectrum of cystine stones (FT−IR: Fourier transform infrared spectroscopic technique).

The chemical composition of stones collected for the litholytic activity was determined using the Fourier transform infrared spectroscopy technique. Figure 1 shows the presence of the main characteristic bands of uric acid (1349 cm^−1^, 1312 cm^−1^, 1122 cm^−1^, 1026 cm^−1^, 992 cm^−1^, and 780 cm^−1^). Figure 2 represents the infra-red spectrum of cystine stones. The main characteristic bands of cystine stones are 1592 cm^−1^, 1492 cm^−1^, 1410 cm^−1^, 1337 cm^−1^, 1193 cm^−1^, 1126 cm^−1^, and 846 cm^−1^.

### 2.2. GC/MS Analysis of the Extracts

The chemical composition of *Argania spinosa* and *Acacia senegal* extracts was determined by comparing the GC/MS spectrum of each peak with the standards obtained from the databases. The identified compounds are shown in Table 1 and Table 2. GC/MS analysis of *Argania spinosa* extract revealed the presence of 17 compounds, including N isobutyl 2,4-undecadiene-8, 10-diynamide, benzoic acid, succinic acid, palmitic acid, and anthraquinone. On the other hand, the GC/MS spectrum of *Acacia Senegal* extract revealed the presence of 14 compounds, among them L-Arabinose, salicylic acid, D Xylose, and Alpha DL-Lyxofuranoside.

### 2.3. In Vitro Dissolution

**Figure 3:**
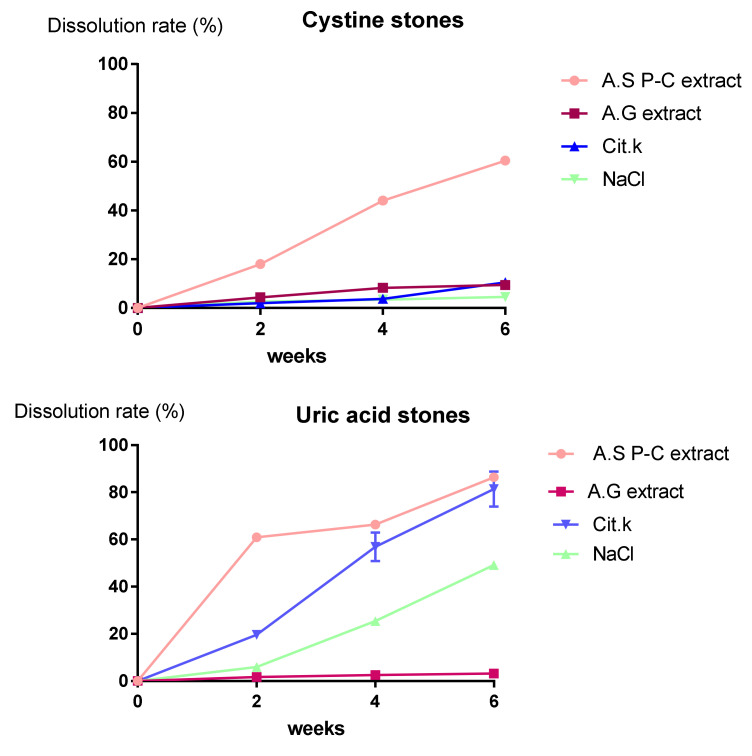
Dissolution rate of A.S P-C (*Argania spinosa* press-cake), A.G (gum arabic), Cit.k (potassium citrate) and NaCl on Cystine and uric acid stones.

The litholytic potential of our plant extracts is illustrated in Figure 3. The result of the litholytic activity of plant extracts on uric acid calculi showed that the extract of *Argania spinosa* had a remarkable dissolving potential (*p* < 0.05) compared with the other extract after two weeks. The mass loss for *Argania spinosa* and gum arabic (*Acacia senegal*) extracts was 23.8 ± 2.12 mg (Dissolution rate (DR) = 60.86 ± 1.48%) versus 1.8 ± 0.85 mg (DR = 1.73 ± 0.1%), respectively. After 6 weeks, the dissolution rate obtained by *Argania spinosa* extract was 86.38 ± 1.06% versus 81.41 ± 7.4% for potassium citrate. Meanwhile, it did not exceed 3.23 ± 0.23% for gum arabic (*Acacia Senegal*) extract. The dissolution rate of NaCl solution was 49.09 ± 1.03%.

**Figure 4:**
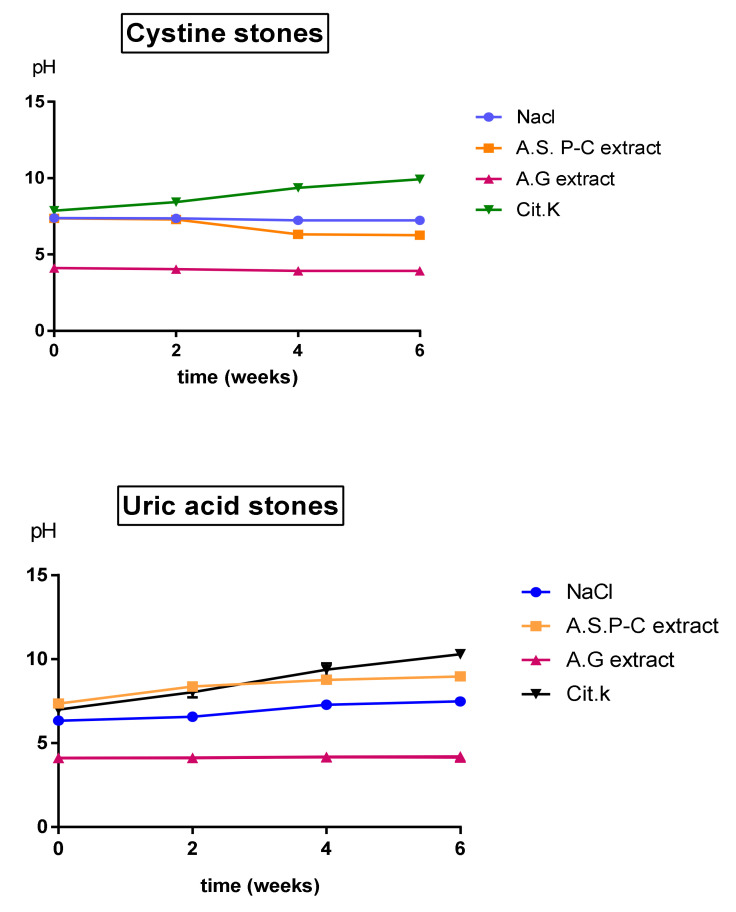
pH measurement of solutions in the presence of cystine and uric acid stones: A.S.P-C—*Argania spinosa*; A.G—*gum Arabic*; Cit.k—potassium citrate.

The kinetic evolution of pH is presented in Figure 4. The pH measurement of uric acid solutions during the 6 weeks showed an acidic pH value for *gum arabic* extract ranging from 4.11 ± 0.006 to 4.16 ± 0.036. The pH of *Argania spinosa* extract and citrate was alkaline, and increased during the 6 weeks from 7.35 ± 0.035 to 8.96 ± 0.015, and from 6.9 ± 0.18 to 10.3 ± 0.17, respectively. The pH value registered in the first week for the physiological solution was 6.33 ± 0.1, and it was 7.48 ± 0.15 in the last week. Concerning the effect of plant extracts on cystine stones, the result of the dissolution test showed that *Argania spinosa* extract had a notable increase (*p* < 0.05) in dissolution rate, with DR = 60.42 ± 1.42%; meanwhile, there was a slight increase for gum arabic extract and citrate, with DR = 9.48 ± 0.12% and DR = 10.51 ± 0.23%, respectively, at the end of the test. The physiological solution induced a weight loss of (4.65 ± 0.31%). The pH measurement indicated that the initial values were different for both plant extracts (7.36 ± 0.05 for *Argania spinosa* and 4.11 ± 0.01 for gum arabic). After the first week of incubation, the pH of *Argania spinosa* and gum arabic solutions underwent a decrease from 7.29 ± 0.06 to 6.27 ± 0.06 and from 4.1 ± 0.01 to 3.93 ± 0.06, respectively. The pH of the citrate solution underwent a linear increase to 9.93 ± 0.15.

### 2.4. In Vitro Calcium Oxalate Crystallization Assay

Stone formation is the outcome of various physicochemical events including nucleation, crystal growth, and crystal aggregation [12]. The most effective method to prevent or to treat urolithiasis is to control the first stage of calculi formation, which is the nucleation event [16].

**Figure 5:**
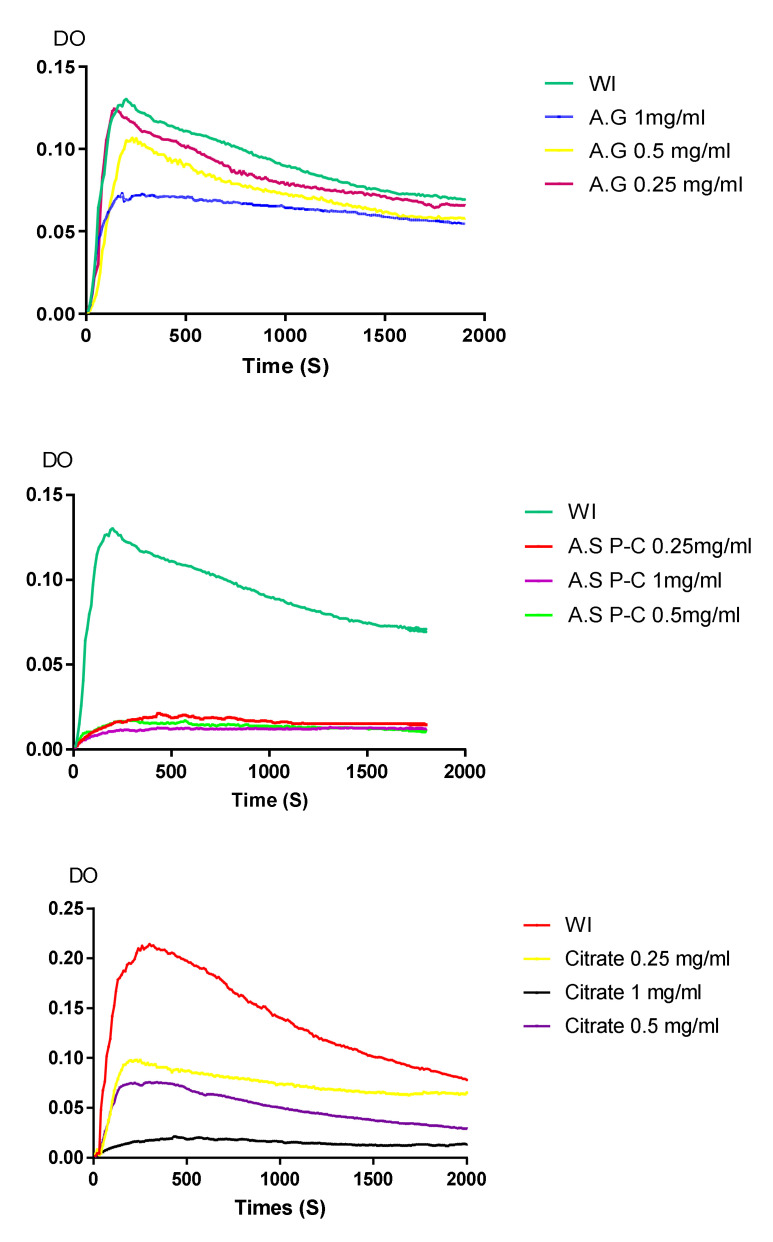
Temporal evolution of the optical density in the presence of A.S P-C (*Argania spinosa*), A.G (*gum Arabic)* extracts, and Citrate. WI—without inhibitor.

In the present work, the inhibitory effect of plant extracts against calcium oxalate nucleation was studied by measuring optical density (OD) at 620 nm. The OD registered without an inhibitor, in the presence of different extract concentrations, and in the presence of potassium citrate is shown in Figure 5. The maximal optical density value (OD max) registered for the test without an inhibitor was 0.138 after 160 s (p) and 0.032 after 560 s (p) for the test in the presence of the *Argania spinosa* extract. Meanwhile, the OD max value for gum arabic extract was 0.126 after 140 s, and 0.031 after 450 s for the positive control (*p* < 0.005 for all comparisons vs. WI). The effect of plant extracts on calcium oxalate crystal formation was determined by calculating the percentage inhibition. To validate our result, the correlation coefficients, as well as the coefficients of variation, were also calculated, and are shown in Table 3. *Argania spinosa* extract had a significant inhibitory effect (*p* ˂ 0.001 vs. Cit.K) for the three concentrations. It inhibited nucleation with a percentage ranging from 83.78 ± 2% to 80.06 ± 2.22%, which is relatively similar to the Cit.K effect, with R > 0.97 and CV ˂ 10%. On the other hand, the percentage inhibition of gum arabic extract did not exceed 43.77 ± 3.82% (*p* ˂ 0.001 vs. Cit.K) for a 1 mg/mL concentration. The 0.5 mg/mL and 0.25 mg/mL concentrations inhibited nucleation, with 27.79 ± 1.6% and 27.22 ± 0.18%, respectively, with R > 0.97 and CV ˂ 10%. The effect of our extract concentrations on the rate of nucleation inhibition revealed a small variation in the effect of *Argania spinosa* extract (R = 0.99; *p* ˂ 0.005). Meanwhile, the rate of nucleation inhibition of gum arabic extract varied from 27.79 ± 1.6 at 0.5 mg/mL to 43.77 ± 3.8 at 1 mg/mL (R = 0.95; *p* ˂ 0.005).

### 2.5. Microscopic Observation

**Figure 6:**
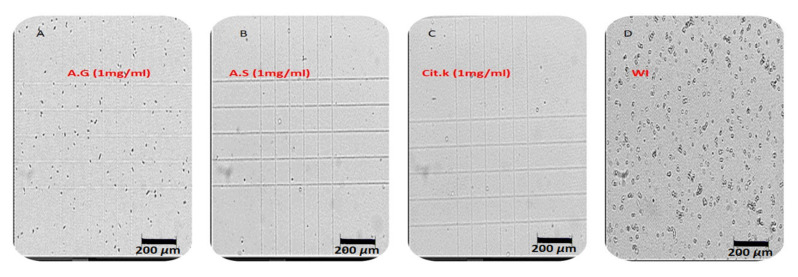
Microscopic observation (400×) of calcium oxalate crystals during the turbidity test with (**A**) gum arabic extract; (**B**) *Argania spinosa* extract; (**C**) potassium Citrate; and (**D**) WI (without inhibitor).

To confirm the inhibitory effect of our extracts, each sample during the experience was observed under a polarized optical microscope (400×) (Figure 6). The microscopic observation showed that in the absence of inhibitors, the number and size of crystals were higher than in the tests with inhibitors. A significant decrease in crystal numbers was observed in the presence of *Argania spinosa* extract and potassium citrate. On the other hand, more numerous crystals were observed in the presence of gum arabic extract.

### 2.6. Characterization of Crystals

**Figure 7:**
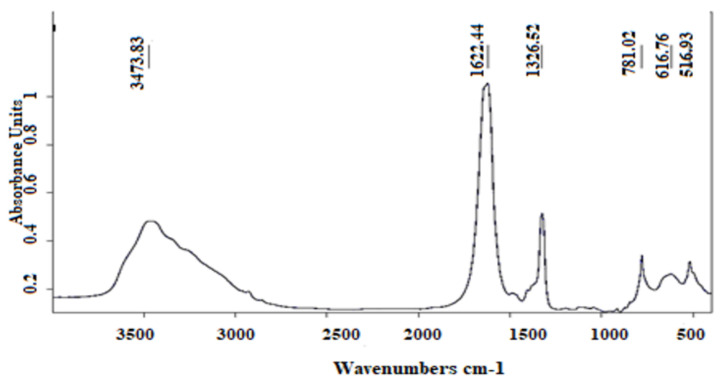
Infrared spectrum of synthesized crystals (FT−IR: Fourier transform infrared spectroscopic technique).

The characterization of crystals using Fourier transform infrared spectroscopy confirmed the composition of synthesized crystals. The infrared spectrum of synthesized crystals is represented in Figure 7. A single absorption peak appeared at 3473 cm^−1^, which corresponds to the COD crystals. The two bands observed at 1622 cm^−1^ and 1326 cm^−1^ correspond to asymmetrical stretching carbonyl (vas (COO−)) and symmetrical stretch band vs (COO−), respectively. Both bands indicate the formation of COM crystals. The absorption peak at 781 cm^−1^ could also indicate the presence of COM, whereas the appearance of the band at 616 cm^−1^ is attributed to COD crystals [17]. The absorption band at 518 cm^−1^ is attributed to pure COM [18].

## 3. Discussion

In the present work, we evaluated and verified the anti-urolithiatic activity of the two plants using two different methods. The first one was the dissolution test of plant extracts on cystine and uric acid stones. The second one was the turbidity test, which aimed to examine the preventive effects on calcium oxalate crystals. Several studies have tested the effects of medicinal plants on cystine stones [19,20], while few studies have focused on testing the dissolution rate of plants on uric acid stones [21]. In our work, the hydro-ethanolic extract of *Argania spinosa* had a significant effect on cystine and uric acid stones. The results obtained for the *gum arabic* extract did not demonstrate any notable effect. The result of the pH measured during the period of incubation for cystine stones showed an increase for Cit.k, whereas the pH of *gum arabic* remained at an acidic value until the end of the test. On the other hand, for the uric acid stones, the pH increased slightly from 7.3 to 8.9 for *Argania spinosa*, and from 6.9 to 8.6 for Cit.k, respectively. The pH of *gum arabic* remained constant at 4.1 throughout the incubation period. This may explain the absence of its dissolution effect on the two types of stones.

For the turbidity test, we used a closed-system model based on temporal measurements of the optical density at 620 nm. In the absence of the inhibitor, the maximal optical density was registered after 160 s. In the presence of *gum arabic* extract, it was registered after 140 s. The induction times for *Argania spinosa* and Cit.k were 560 s and 450 s, respectively. The induction time is an important value to prove nucleation inhibition [22]. This result explains the low effect of *gum arabic* extract (43%) and the significant inhibitory effect of *Argania spinosa* extract (83%) on the nucleation event. Phenolic acids were one of the secondary metabolites contained in *Argania spinosa* (L.) press-cake in our study, and are known for their anti-crystallization properties [23]. Other phytomolecules can contribute and cannot be excluded from anti-urolithiatic activity, such as succinic acid and catechin [20,21,22,23,24,25,26,27,28], which were identified in the *Argania spinosa* extract by GC/MS analysis. In addition, several phenolic acids were identified in the *Argania spinosa* extract. Among them, hydroxybenzoic acid, which was found to be effective in the promotion of antioxidant enzyme expression as well, as in some cardiovascular diseases, by decreasing inflammation that can lead to atherosclerosis [24]. Tetradeca-2E, 4E, 8Etrienoic acid, 4-hydroxyphenylethylamide was another phytochemical identified. It belongs to the alkylamide bioactive compounds, which have antibacterial and antifungal activities as well as antiparasitic activity [25].

L-Arabinose and Salicylic acid were among the phytochemicals identified in the *gum arabic* extract. The first was found to be capable of inhibiting intestinal sucrase activity, and consequently, minimizing postprandial, insulin, glucose, and C-peptide responses [26]. The second was a potent anti-inflammatory and antioxidant agent, which acts through the suppression of prostaglandin synthesis and the decrease of superoxide anion radicals, respectively [27]. In our study, gum arabic extract did not induce any significant effects. On the other hand, the results revealed the effectiveness of *Argania spinosa* extract in the treatment of cystine and uric acid stones, as well as in the prevention of calcium oxalate stone formation. Its richness in various phytochemical compounds could be responsible for its potent antiurolithiatic activity.

## 4. Material and Methods

### 4.1. Plant Materials

Fruits of *Argania spinosa* (L.) Skeels were collected in July 2019 in the Agadir region. The plant’s taxonomic classification was determined. The fruits were peeled and pulped before being used. Nuts were fractured to obtain kernels, which were then mechanically pressed. Argan press-cake was dried at 25 °C and ground into a fine powder with an electric grinder.

Gum arabic was purchased from an herbalist and powdered using a mortar in order to obtain a white powder.

### 4.2. Extraction Method

The extraction followed the method described by Feknous et al. [25] with slight modifications. A total of 100 g of Argania spinosa (L.) Skeels press-cake powder was defatted by 250 mL of n-hexan, stirred for about 24 h, then filtered and dried. The defatted powder and gum arabic powder were subjected to extraction using ethanol/distilled water (60:40 *v*:*v*) and stirred for 24 h, then filtered using a sintered glass Büchner funnel. Ethanol and distilled water were removed using a rotatory evaporator at 40 °C, and the residue was stored at 4°C until any further use.

### 4.3. GC/MS Analysis of the Extracts

For the identification of phytochemical compounds, we followed the method of Kabran et al. [26]. Briefly, 40 g of plants extract powder was treated with petroleum ether, then the two samples of *Argania spinosa* (L.) Skeels press-cake and gum arabic underwent acid hydrolysis using 200 mL of 2N (HCl), and were heated with reflux for two hours. Finally, to the cooled hydrolysate, 3 × 200 mL of ethyl acetate was added.

The organic fractions were dried on anhydrous MgSO_4_ and concentrated under vacuum using a rotatory evaporator, and then dried under nitrogen gas. The organic fraction (100 μL) was derived through the addition of 200 μL of MSTFA (*N*-methyl *N*-trimethylsilyltrifluoroacetamide), then dried and heated to 37 °C for 30 min. An amount of 1 μL of each sample was injected for analysis using gas chromatography coupled with mass spectrometry (GC-MS: Brand Agilent Technologies Model 5973 with an Agilent column 19091S-433 HP-5MS equipped with a single quadrupole mass spectrometer, operated using electron ionization (EI) (Agilent Technologies, Santa Clara, CA, USA)). The chromatographic conditions were as follows: An HP-5MS UI (0.25 mm × 30 m × 0.25 µm) was used. The injector temperature was 300 °C. The carrier gas used was Helium at a flow rate of 0.9 mL/min. The oven temperature was set to 60 °C for 10 min, then increased and held at 300 °C for 20 min. The results obtained were compared with those of the spectral data obtained from the Wiley library.

### 4.4. In Vitro Dissolution

The litholytic activity of our extracts was studied according to the protocol described by Yachi et al. [19], with slight modifications. Uric acid and cystine stones were collected from the nephrology service of the University Hospital Center Hassan II Fez, Morocco. They were examined morphologically using a binocular stereo microscope. The chemical composition of each sample stone was studied using Fourier transform infrared spectroscopy analysis (FT-IR) (BRUKER OPTICS GMBH & CO.KG. Ettlingen, Germany). An amount of 0.5% of plant extracts were prepared in physiological solution (9 g/L of NaCl). Stones were weighed and placed in a porous bag, then placed in suspension under stirring for 6 weeks in an Erlenmeyer glass containing extract solutions. All contact with the magnetic stirring bar was avoided. To measure the mass loss, calculi were removed every week, dried for 18 h at a temperature of 40 °C, weighed with a precision balance, and then delivered to the Erlenmeyer glass. The pH of solutions was also measured. Potassium citrate was used as a positive control at a concentration of 3 mmol/L. An amount of 0.9% of physiological solution was used as a negative control. Each experiment was conducted in triplicate.

The dissolution rate (DR%) was obtained by comparing the initial mass before incubation and the residual weight using the following formula:

DR% = (W_initial_ − W_final_) × 100/W_initial_

W _initial_: weight of calculi before incubation with plant extracts;

W _final_: weight of calculi after incubation with plant extracts.

### 4.5. In Vitro Calcium Oxalate Crystallization Assay

#### 4.5.1. Inhibition Test Using Turbidity

The inhibition of CaOx crystallization was studied using a turbidimetric method, according to the method of Sweta et al. [28], with modifications of some parameters. Stock solutions of solution A: sodium oxalate Na_2_C_2_O_4_ (1 mM), and solution B: calcium chloride CaCl_2_ (8 mM) containing 200mM NaCl were prepared. Both solutions were filtered using micro-filter (0.45 μm) and then warmed to 37 °C until starting the test. The stock solutions were freshly prepared each day, and the pH was adjusted to 5.7 by adding sodium acetate (10 mM).

Calcium oxalate crystallization was induced without an inhibitor by mixing solutions A and B; Amounts of 1 mL of CaCl_2_ solution and 1ml of distilled water were transferred to a quartz cuvette, and an identical volume of Na_2_C_2_O_4_ was added to initiate crystal formation. The mixed solutions were maintained at 37 °C under a stirring circulating system. Every 10 s for the first ten minutes and every thirty seconds for the remaining time, temporal measurements of the optical density ‘‘OD’’ were registered at a 620 nm wavelength for 30 min using an ultraviolet–visible spectrophotometer (Labtron Equipment Ltd., Camberley, UK). The crystallization assay with inhibitors was carried out using three concentrations ranging from 0.25 mg/mL to 1 mg/mL of plant extracts containing physiological solution (200 mM). Extract solutions were added to CaCl_2_ solution, then the same volume of Na_2_C_2_O_4_ was added and the ‘‘OD’’ was measured as described above. Each experiment was performed in triplicate. The percentages of inhibition were calculated using the following formula:

% I = (1 − absorber of test with inhibitor/absorber of test without inhibitor) × 100

#### 4.5.2. Microscopic Observation

During each experiment, and after the induction of crystal formation, a sample was observed under a polarized optical microscope (400×) connected to a computer and equipped with a digital camera. The tests were performed as independent triplicates for each extract concentration.

#### 4.5.3. Characterization of Crystals

Synthesized crystals were characterized using Fourier transform infrared spectroscopy (Brucker). A volume of solution A (8 mM CaCl_2_·2H_2_O) and an equal volume of solution B (1 mM Na_2_C_2_O_4_) were mixed and stirred continuously for 30 min and stored at 37 °C for 24 h. Then, the precipitate was dried until examination [29].

### 4.6. Data Analysis

The results were expressed as mean ± SD. The statistical analyses were carried out using a one-way ANOVA followed by Tukey’s multiple comparison test and Pearson’s correlation using GraphPad Prism 7; *p* < 0.05 was considered significant.

## 5. Conclusions

The use of medicinal plants for the treatment of various diseases has become an alternative treatment because of its minimal side-effects. This work is devoted to the verification of the antiurolithiatic effect of two different plants: *Argania spinosa* (L.) press-cake and *Acacia senegal* (L.) Willd. Our outcomes revealed that *Argania spinosa* extract possessed remarkable litholytic activity on cystine and uric acid stones compared to *gum arabic* extract. Additionally, the effect of the studied extracts on calcium oxalate crystallization showed that the percentage of nucleation inhibition was in the order of 83.78% and 43.77% for *Argania spinosa* press-cake (L.) and *Acacia senegal* (L.) Willd., respectively. The identified phytochemicals in the *Argania spinosa* (L.) press-cake were especially dominated by phenolic acids, which could be responsible for the antiurolithiatic activity.

## Figures and Tables

**Table 1:** Chemical composition of *Argania spinosa* extract.

Peak	RT (min)	*m*/*z* Quasi-Molecular Peak	Compounds	Chemical Formula
1	4.34	168 (M + H)^+^	methyl 3,4-dihydroxybenzoate	C_8_H_8_O_4_
2	4.92	290 (M)^+^	Epicatechin	C_15_H_14_O_6_
3	5	150 (M)^+^	4-ethylbenzoic acid	C₉H_10_O_2_
4	6.13	241 (M)^+^	Sarcosine, *N*-(trifluoroacetyl)-, butyl ester	C₉H₁₄F₃NO₃
5	6.98	90 (M)^+^	Lactic acid	C_3_H_6_O_₃_
6	7.71	90 (M)^+^	Ethandioic acid, bis (trimethyl silyl) ester §§ Oxalic acid	C_2_H_2_O_4_
7	8.44	248 (M + H)^+^	Propanedioic acid, bis (trimethylsilyl) ester §§ Malonic acid bis trimethylsilyl ester.	C_9_H_20_O_4_Si_2_
8	9.38	262 (M)^+^	Butanedioic acid, bis (trimethylsilyl) ester §§ succinate. (Succinic acid) (Bis trimethylsilyl succinic acid).	C_10_H_22_O_4_Si_2_
9	9.62	260 (M + H)^+^	2-Butanedioic acid(*E*)-bis (trimethylsilyl) ester §§ Fumaric acid, bis trimethylsilyl ester.	C_10_H_20_O_4_Si_2_
10	10.63	248 (M + H)^+^	Malonic acid (propane dioic acid, bis (trimethylsilyl ester).	C₉H₂₀O₄Si₂
11	10.75	350 (M + H)^+^	Malic acid, *O*-trimethylsilyl, bis (trimethylsilyl) ester	C_13_H_30_O_5_Si_3_
12	12.61	208 (M)^+^	Anthraquinone, 1-(2,6-xylyl)-(CAS)	C_14_H_8_O_2_
13	12.77	122 (M)^+^	Benzoic acid	C7H6O2
14	12.88	331 (M + H)^+^	2,6, Bis (2-naphthyl) Pyridine	C_25_H_17_N
15	14.11	256 (M)^+^	Palmitic acid	C₁₆H₃₂O₂
16	15.18	341 (M)^+^	Tetradeca-2*E*,4*E*,8*E*trienoic acid 4-hydroxyphenylethylamide	C₂₂H₃₁NO₂
17	17.96	96 (M)^+^	Silicic acid	H₄O₄Si

**Table 2:** Chemical composition of *Acacia senegal* extract.

	RT (min)	*m/z* Quasi-Molecular Peak	Compounds	Chemical Formula
1	4.95	194 (M + H)^+^	D-Glucuronic acid	C_6_H_10_O_7_
2	6.72	-	None identified	-
3	9.26	92 (M)^+^	Glycerol	C_3_H_8_O_3_
4	9.37	150 (M + H)^+^	L-Arabinose	C_5_H_10_O_5_
5	11.72	150 (M + H)^+^	D-Xylose	C_5_H_10_O_5_
6	11.67	164 (M)^+^	Alpha-DL-Lyxofuranoside	C_6_H_12_O_5_
7	11.93	150 (M + H)^+^	D-ribose	C_5_H_10_O_5_
8	13.75	281 (M + H)^+^	2-methoxy(1)benzothienol(2,3-c)quinolin-6(5*H*)-one	C_16_H_11_NO_2_S
9	14.14	256 (M)^+^	Palmitic acid	C₁₆H₃₂O₂
10	16.65	-	None identified	-
11	16.95	152 (M + H)^+^	Mandelic acid	C_8_H_8_O_3_
12	17.74	138 (M + H)^+^	Salicylic acid	C_7_H_6_O_3_
14	17.96	346(M + H)^+^	Gibberellin A3 (Gibb-3-ene-1,10-dicarboxylic acid,2,4,a,7 trihydroxy)	C_19_H_22_O_6_

**Table 3:** Percentage inhibition of nucleation in the presence of plant extracts and Citrate.

Concentration (mg/mL)	% of Inhibition			R			CV		
	A.S	A.G	Cit.k	A.S	A.G	Cit.k	A.S	A.G	Cit.k
1	83.78 ± 2	43.77 ± 3.82	84.6 ± 6.03	0.96	0.98	0.95	2.39	8.72	7.13
0.5	81.26 ± 4.2	27.79 ± 1.6	83.2 ± 1	0.97	0.95	0.98	5.17	5.77	1.2
0.25	80.06 ± 2.2	27.22 ± 0.18	78.32 ± 4.4	0.98	0.98	0.99	2.77	0.68	5.71

Values are expressed as mean ± SD (*n* = 3). A.S: hydroalcoholic extract of *argania Spinosa* (L.) press-cake; A.G: hydroalcoholic extract of gum arabic; Cit.K: potassium citrate. R: correlation coefficient, CV: variation coefficient.

## Data Availability

Not applicable.

## Sample Availability

Samples are available from the authors upon reasonable request.

## References

[1] Daudon M., Bader C.A., Jungers P. (1993). Urinary Calculi: Review of Classification Methods and Correlations with Etiology. Scanning Microsc..

[2] Miller N.L., Lingeman J.E. (2007). Management of Kidney Stones. BMJ.

[3] York N.E., Borofsky M.S., Lingeman J.E. (2015). Risks Associated with Drug Treatments for Kidney Stones. Expert Opin. Drug Saf..

[4] Hebi M., Khallouki F. (2022). Cardiovascular & Hematological Agents in Medicinal Chemistry. Curr. Med. Chem. Cardiovasc. Hematol. Agents.

[5] El Monfalouti H., Guillaume D., Denhez C., Charrouf Z. (2010). Therapeutic Potential of Argan Oil: A Review. J. Pharm. Pharmacol..

[6] El Monfalouti H., Charrouf Z., Belviso S., Ghirardello D., Scursatone B., Guillaume D., Denhez C., Zeppa G. (2012). Analysis and Antioxidant Capacity of the Phenolic Compounds from Argan Fruit (*Argania Spinosa* (L.) Skeels). Eur. J. Lipid Sci. Technol..

[7] Guillaume D., Charrouf Z. (2005). Saponines et métabolites secondaires de l’arganier (*Argania spinosa*). Cah. Agric..

[8] Elshama S.S. (2018). The Preventive Role of Arabic Gum in the Treatment of Toxicity. Open Access.

[9] Elamin S., Alkhawaja M.J., Bukhamsin A.Y., Idris M.A.S., Abdelrahman M.M., Abutaleb N.K., Housawi A.A. (2017). Gum Arabic Reduces C-Reactive Protein in Chronic Kidney Disease Patients without Affecting Urea or Indoxyl Sulfate Levels. Int. J. Nephrol..

[10] Taïbi K., Aït Abderrahim L., Boussaid M., Taibi F., Achir M., Souana K., Benaissa T., Farhi K.H., Naamani F.Z., Nait Said K. (2021). Unraveling the Ethnopharmacological Potential of Medicinal Plants Used in Algerian Traditional Medicine for Urinary Diseases. Eur. J. Integr. Med..

[11] Jaradat N.A., Zaid A.N., Al-Ramahi R., Alqub M.A., Hussein F., Hamdan Z., Mustafa M., Qneibi M., Ali I. (2017). Ethnopharmacological Survey of Medicinal Plants Practiced by Traditional Healers and Herbalists for Treatment of Some Urological Diseases in the West Bank/Palestine. BMC Complement. Altern. Med..

[12] El Oumari F.E., Bousta D., Grafov A., Sqalli Houssaini T. (2021). Phytomolecules Investigated for the Prevention and Treatment of Urinary Stones. Mediterr. J. Chem..

[13] Cienfuegos-Pecina E., Ibarra-Rivera T.R., Saucedo A.L., Ramírez-Martínez A., Esquivel-Figueroa D., Domínguez-Vázquez I., Alcántara-Solano K.J., Moreno-Peña D.P., Alarcon-Galvan G., Rodríguez-Rodríguez D.R. (2020). Effect of Sodium (*S*)-2-Hydroxyglutarate in Male, and Succinic Acid in Female Wistar Rats against Renal Ischemia-Reperfusion Injury, Suggesting a Role of the HIF-1 Pathway. PeerJ.

[14] Kasarci G., Ertugrul B., Iplik E.S., Cakmakoglu B. (2021). The Apoptotic Efficacy of Succinic Acid on Renal Cancer Cell Lines. Med. Oncol..

[15] Akomolafe S.F., Akinyemi A.J., Anadozie S.O. (2014). Phenolic Acids (Gallic and Tannic Acids) Modulate Antioxidant Status and Cisplatin Induced Nephrotoxicity in Rats. Int. Sch. Res. Not..

[16] Ram J., Moteriya P., Chanda S. (2015). An Overview of Some Promising Medicinal Plants with in Vitro Anti-Urolithiatic Activity. IOSR J. Pharm..

[17] Yao X.-Q., Ouyang J.-M., Peng H., Zhu W.-Y., Chen H.-Q. (2012). Inhibition on Calcium Oxalate Crystallization and Repair on Injured Renal Epithelial Cells of Degraded Soybean Polysaccharide. Carbohydr. Polym..

[18] Chen J.-Y., Sun X.-Y., Ouyang J.-M. (2020). Modulation of Calcium Oxalate Crystal Growth and Protection from Oxidatively Damaged Renal Epithelial Cells of Corn Silk Polysaccharides with Different Molecular Weights. Oxidative Med. Cell. Longev..

[19] Feknous S., Saidi F., Said R.M. (2014). Extraction, caractérisation et identification de quelques métabolites secondaires actifs de la mélisse (*Melissa officinalis* L.). Nat. Technol..

[20] Kabran G.R., Mamyrbekova-Bekro J.A., Pirat J.-L., Bekro Y.-A., Sommerer N., Verbaere A., Meudec E. (2014). Identification de composés phénoliques extraits de deux plantes de la pharmacopée ivoirienne. J. Société Ouest-Afr. Chim..

[21] Yachi L., Bennis S., Aliat Z., Cheikh A., Idrissi M.O.B., Draoui M., Bouatia M. (2018). In Vitro Litholytic Activity of Some Medicinal Plants on Urinary Stones. Afr. J. Urol..

[22] Hennequin C., Lalanne V., Daudon M., Lacour B., Drueke T. (1993). A New Approach to Studying Inhibitors of Calcium Oxalate Crystal Growth. Urol. Res..

[23] El Menyiy N., Khouchlaa A., El Omari N., Zengin G., Gallo M., Montesano D., Bouyahya A. (2021). Litholytic Activities of Natural Bioactive Compounds and Their Mechanism Insights. Appl. Sci..

[24] Juurlink B.H., Azouz H.J., Aldalati A.M., AlTinawi B.M., Ganguly P. (2014). Hydroxybenzoic Acid Isomers and the Cardiovascular System. Nutr. J..

[25] Boonen J., Bronselaer A., Nielandt J., Veryser L., De Tre G., De Spiegeleer B. (2012). Alkamid Database Chemistry, Occurrence and Functionality of Plant N-Alkylamides. J. Ethnopharmacol..

[26] Krog-Mikkelsen I., Hels O., Tetens I., Holst J.J., Andersen J.R., Bukhave K. (2011). The Effects of L-Arabinose on Intestinal Sucrase Activity: Dose-Response Studies in Vitro and in Humans. Am. J. Clin. Nutr..

[27] Randjelović P., Veljković S., Stojiljković N., Sokolović D., Ilić I., Laketić D., Randjelović D., Randjelović N. (2015). The Beneficial Biological Properties of Salicylic Acid. Acta Fac. Med. Naissensis.

[28] Bawari S., Negi Sah A., Tewari D. (2018). Antiurolithiatic Activity of Daucus Carota: An In Vitro Study. PeerJ.

[29] Zhang Y., Tang Y., Xu J., Zhang D., Lu G., Jing W. (2015). Modulation of Polyepoxysuccinic Acid on Crystallization of Calcium Oxalate. J. Solid State Chem..

